# Electromagnetic Vibrational Energy Harvesters: A Review

**DOI:** 10.3390/s22155555

**Published:** 2022-07-25

**Authors:** Andrew Muscat, Soham Bhattacharya, Yong Zhu

**Affiliations:** 1 School of Engineering and Built Environment, Griffith University, Nathan, QLD 4111, Australia; andrew.muscat@alumni.griffithuni.edu.au; 2 Department of Electrical and Computer Engineering, Rowan University, Glassboro, NJ 08028, USA; sohambhattacharya36@gmail.com

**Keywords:** electromagnetic, vibration, energy harvesters, wireless sensor nodes (WSN), microelectromechanical systems (MEMS), renewable energy, Internet of things (IoT)

## Abstract

As industries need more real-time monitoring and interconnected systems, the demand for wireless sensors expands. Vibrational energy harvesters are a potential solution for powering these sensors, as vibrations commonly exist where monitoring occurs. Developments in low-power circuitry have also led to the feasibility of these types of harvesters. Electromagnetic harvesters are a standout among various types of vibrational harvesters due to their ability to capture kinetic energy in a low-frequency range. This leads to these devices being more applicable in real-world applications where ambient vibrations are typical of having low frequencies. Hence, extensive research has been undertaken to make electromagnetic harvesters more efficient and compact. This review study aims to examine recent literature that has made advancements and demonstrated the full potential of such devices.

## 1. Introduction

In the last decade, extensive research has been carried out on the development of vibrational energy harvesters for Internet of things (IoT). This is due to advancements in ultra-low power (ULP) circuits, as well as the need for wireless sensing units [1]. Current wireless sensor nodes (WSN) typically use an electrochemical battery to power them. However, conventional batteries generally have a limited lifetime of up to 15 years when drawing currents in the µW range [2]. Some applications for these WSN lead to their battery replacement being either too difficult or too costly [3].

Renewable energy sources, such as UV radiation, thermal heat, and wind power, are well understood and applicable in outdoor environments. However, the majority of these are highly dependent on weather conditions and generate significantly less power when operated indoors [4,5]. This leads to vibrational energy harvesters being an advantageous power source alternative due to the large ambience of vibrations in the real world. Vibrational energy harvesters create energy by converting mechanical vibrations into electricity. These harvesters are typically grouped into piezoelectric, electrostatic, and electromagnetic categories according to their working principles. Each of these transducers has its own drawbacks and advantages.

Technologies for piezoelectric vibrational energy harvesting have recently received a lot of attention, and harvesters have been successfully used in a variety of sectors, including architecture, biomechanics, and human motion. Piezoelectric harvesters work off the piezoelectric effect, where a strain in a material leads to deformation of the structure, causing an imbalance in charge and thus producing a voltage [6]. Piezoelectric energy harvesters obtain the electric energy generated when these piezoelectric materials are vibrated [7]. They typically need to be operated at a high frequency (>1 kHz), which limits their kinetic energy harvesting capability, as ambient vibrations are usually on the scale of 1–100 Hz [8]. Piezoelectric harvesters have the advantages of self-powering, relatively high output voltage, compact size, and a high electromechanical coupling coefficient. However, they are subject to the adverse effect of piezoelectric materials producing varying outputs throughout their operational life [9] and can even totally fail due to brittle material fatigue [10]. Beeby et al. showed that while compressive strain piezoelectric materials can offer better material longevity, the nature of the required strain limits where and how they can be applied [11].

Electrostatic transducers work by a force, creating a change in capacitance, leading to voltage induction [12]. The electrostatic methodology comprises electret-type vibrational energy harvesting with MEMS, as well as triboelectric energy harvesting [13]. Electrostatic harvesters inherently require a high-voltage power supply or electret to build strong electric fields to push the electric current move, which makes the system complicated [14]. Additionally, considering that the changes in plate separation or area are typically in the mm range, they are less suitable for larger amplitude vibrations (as would be expected from human movement) without additional system complexity to gear the input environmental motion to a suitable scale [11].

Electromagnetic vibrational energy harvesters (EVEH), on the other hand, have a relatively simple construction and generate sound power at low frequencies, so they have received significant attention [1,15]. Electromagnetic harvesters use the principle of Faraday’s law of induction in which a magnet passing through a coil induces a current [16]. Electromagnetic induction and inverse magnetostrictive effects are commonly adopted for electromagnetic energy conversions. In the inverse magnetostrictive method, the magnetization state of a magnetostrictive material is controlled by applying a bias magnetic field to the material using permanent magnets, followed by applying a strain to the material to generate a change in magnetic flux, which is converted into electric power using a coil [17,18]. In [19], the authors evaluated key magnetically coupled piezoelectric vibration energy harvesting technologies and assessed the possible advantages of magnetic force on these technologies. According to their various structural properties, they are divided into five groups: monostable, bistable, multi-stable, magnetic plucking, and hybrid piezoelectric-electromagnetic energy harvesters. This review examines the research in the literature that has been conducted to develop electromagnetic harvesters in recent years. Section 2 summarizes the underpinning electromagnetic principles for kinetic to electrical energy conversions. Section 3 and Section 4 delve into the design modifications introduced to enhance power and efficiency. Section 5 reviews the various technologies used for fabrication and the structures used, while Section 6 provides insights into the circuitry used in real-world applications. Section 7 and Section 8 provide an overview of the literature and this review, respectively.

## 2. Electromagnetic Vibrational Energy Harvesting Principles

### 2.1. Electromagnetic Theory

In 1831, Michael Faraday discovered that when a wire and magnet move relative to one another, the cutting of the magnetic flux results in a current being induced to the wire, in turn producing a voltage. The amount of voltage that can be produced depends on the number of loops in the coil and the rate of change in the magnetic flux [1,20]. This principle is summarized by Faraday’s law:

(1) $$\varepsilon =-N\frac{\Delta \phi }{\Delta t}$$

where, *ε* is the voltage produced in terms of EMF, *N* is the number of loops of the coil, and *φ* is the magnetic flux. A negative sign arises due to Lenz’s law.

The above formula can be broken down further by investigating the rate of change in magnetic flux. This leads the equation to become:

(2) ε=βlv

where, *β* is the strength of the magnetic field, *l* is the length of the wire, and *v* is the relative velocity between the magnet and the wire [21]. Implicated in Equation (2), to increase the generated voltage, magnetic field, wire length, and relative velocity are the key factors that must be increased.

In a mass-spring-damper-based electromagnetic generator (either a moving magnet or moving coil configuration), the maximum harvested power is [22]:

(3) $$P_{max}=\frac{mY_{0}^{2}\omega^{3}}{4\zeta }$$

where *m* is the movable structure’s mass in the harvester. *ζ* is the transducer damping factor (depending on the transducer impedance). *Y*_0_ and *ω* are the vibration amplitudes and frequencies from the environment, respectively. To maximize harvested power, the damping factor should be low, and the natural frequency of the seismic suspension of the micro generator should be equal to the vibration frequency of the source.

When an electromagnetic energy generator delivers energy to an electrical load, the maximum electrical power is extracted when the electrical damping is equal to the parasitic mechanical damping [23]. In the case where parasitic damping is much greater than electromagnetic damping, the optimum load resistance becomes coil resistance.

### 2.2. Vibration Frequency Considerations

Another factor that dictates an electromagnetic harvester’s potential usage is its resonance frequency. As most harvesters rely on suspension systems, whether a coil or magnet supported by a spring or magnetic levitation, they act as a spring-mass-damper system [24]. This leads to harvesters being considered as a 2nd order system in which they have a resonance frequency [25,26]. A harvester will have spikes in voltage when excited by an input with the same frequency as the resonance [27]. The resonance frequency can be altered by adjusting the dampening of the system, the weight of the proof mass, or the spring constant [28]. Ibrahim et al. [29] described a vibration-based electromagnetic energy harvester whose resonance frequency can be tuned to match the excitation frequency. The frequency was adjusted by controlling a rotatable arm with tuning masses at the tip of a cantilever-type energy harvester, thereby changing the system’s effective mass moment of inertia. The rotatable arm was mounted on a servomotor that was autonomously controlled by a microcontroller and a photosensor to maintain resonance for maximum power generation. To predict the system response for different design parameters and estimate the generated power, a mathematical model was developed. A distributed parameter model was used to examine the system’s natural frequency variation and dynamic response. The analytical model was then validated experimentally by tuning the frequency from 8 Hz to 10.25 Hz.

To maximize the harvested energy, vibrations at different frequencies need to be included in the harvesting system. Some designs have tried to increase the bandwidth of energy harvesting by placing an array of harvesters with different resonance frequencies. However, these are bulky and have a low power density. Liu et al. [30] was able to develop an MEMS harvester that had at least 9 resonance frequencies over a frequency range of 100 Hz to 800 Hz. The harvester was only able to produce voltages ranging from 0.01 mV to 0.13 mV. However, it was the first MEMS device capable of achieving nine resonant peaks for its size. The use of multiple cells was investigated by Liu et al. [31]. The MEMS device was able to harvest vibrations from 3-dimensional excitation. The device utilized 3 coils mounted to a circular structure with the capability to flex in any direction. Due to this, the harvester had 3 resonance frequencies of 1285, 1470, and 1550 Hz. Marin et al. [32] constructed a traditional mechanically fabricated harvester in which it had 2 cells for power harvesting. The design used wound coils attached to cantilevers with magnets arranged around the coils. The prototype was compared to a single-cell harvester of a similar design. The double cell saw an increase in power density of 66%.

To power wireless sensor nodes for bridge health monitoring, ref. [33] offered unique electromagnetic bridge energy harvesters (BEHs), which have multiple resonant frequencies. The broadened frequency band increases the energy harvesting efficiency from wind surges and bridge vibrations. The created BEHs are cantilever-type devices made up of a support, an airfoil, a cantilever beam, a wrapped coil, and a permanent magnet. Harvesters are evaluated in a lab setting with varying vibration levels and air surges of varying speeds.

Many researchers have investigated mechanical frequency up-converting techniques as a means of improving the harvester’s bandwidth performance. The idea behind the mechanical frequency up-converting technique is to convert a low-frequency input signal into higher-frequency signals. This has been achieved by using mechanical cantilevers, which, when excited, are vibrated at their natural frequency. This is preferable, as a high frequency will provoke more flux to be cut, or higher velocity *v* in Equation (2), leading to higher power output [8]. Klein and Zuo [34] constructed a harvester for the purpose of its use in nuclear power plants. Their design used a flat spring structure that was able to capture low-frequency vibrations and transform them into higher frequencies. It was able to produce a voltage of 910 mV and a power of 2 mW. Zorlu et al. [35] used a cantilever that was held by a mechanical barrier composed of a membrane. When enough acceleration was applied to the cantilever, it was able to move away from the membrane and operate at its own frequency. The device was able to turn an initial vibration of 10 Hz into 394 Hz. From theoretical analysis and prototyping investigation, it was determined that this kind of structure is a feasible design for scaling down. It was hypothesized that the power density of the device would increase with miniaturization.

Another attempt to broaden the output bandwidth of the system is a multi-stable electromagnetic harvester. In [36], Yang et al. proposed a theoretical model and dynamical analysis of a novel multi-stable energy harvester employing a geometric nonlinearity technique. The energy harvester has multiple stable potential energy functions, ranging from mono-stable to quad-stable, by varying the geometric nonlinearity parameters. Therefore, the results demonstrate that such a harvester outperforms traditional linear harvesters. In [37], Kim et al. investigated the dynamic and energetic properties of a multi-stable bimorph cantilever energy harvester that makes use of the magnetic attraction effect. The magnetic field produced by the external magnets tends to have a significant impact on the magnetic force and moment applied to the cantilever tip.

## 3. Single-Magnet versus Multiple-Magnet Structures

From Equation (2), a clear parameter for increasing the output voltage is the strength of the magnetic field, β. As most designs implement neodymium magnets (NdFeB), variations in prototypes have been made by interchanging the number of magnets used in the harvester. By having several magnets, the rate at which the magnetic flux is cut can also be higher due to the larger number of poles. However, introducing more magnets into a design can lead to a higher amount of volume used and a lower power density.

### 3.1. Single Magnet Structures

**Figure 1:**
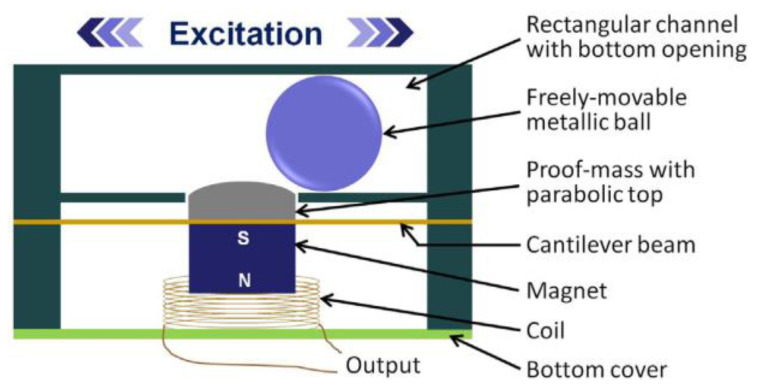
A photo of the harvester created by [38]. A ball rolls in the top section, causing depressing of the cantilever, leading to the magnet being pushed through the coil.

Single magnet electromagnetic harvesters are commonly known for their small size. However, Ref. [38] designed and prototyped a novel harvester that used a spherical ball to transverse a cantilever, causing deflection and a magnet attached to the cantilever to be pushed down through a coil, as shown in Figure 1. Due to the nature of the design, the device had a low resonance frequency of 5.8 Hz but also a relatively large volume of 19.2 cm^3^. This volume led the design to have an unfavorable power density of 5.4 µW/cm^3^.

In [39], an MEMS device was proposed utilizing a single moving magnet mounted on a planar spring with a silicon substrate. The harvester had a total volume of 0.13 cm^3^. When excited at 55 Hz, it was able to achieve a power output of 0.61 µW. This leads the harvester to have a power density of 4.69 µW/cm^3^, which is low compared to most other harvesters. However, this was the smallest harvester investigated and shows that research is being undertaken to improve the miniaturization of these devices. Single magnets are commonly used in MEMS-sized harvesters, as magnets largely make up the size of these harvesters.

Saha et al. [40] developed a traditional EVEH by levitating a single magnet between two magnets. Its purpose was to generate power by walking and running slowly. When excited at a frequency of around 8 Hz at 0.38 g, the design was capable of producing 0.3–2.46 mW of power. Although these findings are considered noteworthy, the device had a relatively large volume of 12.7 cm^3^. As a result, many researchers have employed multiple magnet arrays to reduce device size while increasing the output power generated by mechanically fabricated devices.

### 3.2. Multiple Magnet Structures

Pancharoen et al. [41] investigated human motion as a technique for harvesting vibrational energy. They prototyped a harvester with a volume of just 2.26 cm^3^ with the intention of using it as a power source for joint replacement monitoring. They conducted two experiments: the first with a two-magnet sandwich and the second with an eight-magnet array. Using an array structure rather than a sandwich structure, the harvester was able to achieve a 160% increase in voltage and a 484% increase in power while conducting the running experiments.

**Figure 2:**
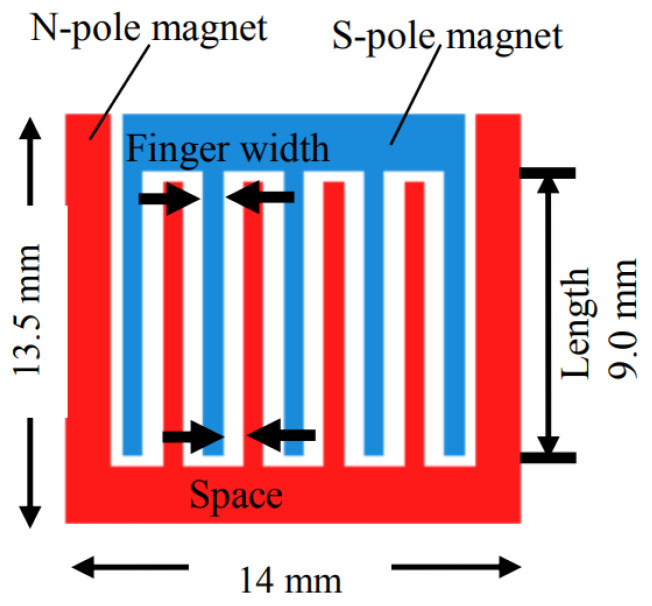
The proposed design for the magnet arrangement for the MEMS harvester created by [42].

Meanwhile, Yamaguchi et al. [42] fabricated an EVEH using MEMS techniques, as illustrated in Figure 2. They were able to create an array using NdFeB film, which was cut into a comb formation. The estimated power was 3.12 µW with a comb-finger width of 40 µm at a frequency of 400 Hz. More noteworthy, they found that a decrease in the magnetic flux density would occur when the single-direction monopolar magnetic structure was too tightly placed. The authors concluded that this was due to the interaction of the fields causing a “smoothing effect”.

It is evident that research has taken place to investigate the number of magnets that should be used to create a more compact and efficient harvester. As shown by [42], too closely packed magnets can drastically change the harvester’s performance. As a result, more developments have been studied using Halbach arrays. Halbach arrays (HA) are an array of permanent magnets arranged to produce a very strong magnetic field on one side, while the other is practically canceled. This property makes HA a viable choice in EVEH construction, as it can increase the harvested power and decrease volume. They also limit electromagnetic interference to other electronics near the harvester, such as sensors and power management circuits [43].

Liu et al. [43] produced an EVEH comprised of a moving coil mounted to a cantilever as well as fixed Halbach arrays mounted on either side of the coil. The design allows the resonance frequency to be easily modified by adjusting the length of the cantilever and hence the tracks in the aluminum holder. This system was able to produce an optimal peak-to-peak voltage of 21.2 V at 11.2 Hz under an acceleration of 0.5 g. This design showed true promise, as its normalized power density is 5.56 mW/cm^3^/g^2^.

In a set of experiments conducted by Zhu et al. [44], the effects of normal magnet layouts were compared to Halbach arrays, as well as the number of magnets and the number of arrays used. The authors conducted experiments in which a single HA and a double HA were trialed against normal sandwich magnet layouts of 4 and 7 magnets. From the theoretical results, using double HA and triangular cross-section magnets could improve the changing rate of magnetic flux by 1.88 and 2.74 times, respectively, compared to the singular standard HA. From the experimental results, the triangular HA was able to increase the output power by 350% compared to the standard HA. This is the only literature found that has investigated the potential of triangular cross-sectional Halbach arrays. Although this is the case, later experiments found that the triangular HA did not perform as well as the normal 4 and 7 magnet layouts. Meanwhile, the double array was able to increase power by 700%. This astonishing figure shows the real potential for the development of multiple HA vibrational harvesters.

Finding a balance between the number of magnets and the size of the EVEH is obvious, and this balance must be achieved to obtain optimum efficiency. The literature suggests that using multiple magnets in arrays is advantageous when designing an EVEH.

It can be concluded from the literature that multiple magnets in arrays are advantageous in the design of an EVEH over a single magnet counterpart. It is evident that finding a balance between the number of magnets and the size of the EVEH is critical in designing a harvester, and this balance must be achieved to reach acceptable power efficiency.

## 4. Moving Coil vs. Moving Magnet

Electromagnetic harvesters are commonly divided into two groups: moving coils and moving magnets [45,46]. Each group allows for specific advantages and disadvantages, leading to their choice being important for the desired properties of the harvester. A moving coil structure uses the coil as a proof mass. As stated earlier, a heavier proof mass results in a lower resonance frequency. To achieve a low resonance frequency, many researchers opt for a coil with a very high number of turns. From Equations (1) and (2), it is clear that this leads to a high-voltage output. Although this result is optimal, a moving coil usually results in a large volume. Moving magnets, on the other hand, have a highly customizable dampening coefficient. This is due to their design being able to have spring, cantilever, magnetic levitation, or ferrofluid for their suspension [47].

### 4.1. Moving Magnet Structure

In moving magnet systems, the coil is placed near the moving magnet or with the magnet moving inside the coil. In the latter, a very compact design can be achieved. In [48], Khan et al. achieved a non-linear vibrational harvester using microelectromechanical systems (MEMS) technologies. The harvester had a polydimethylsiloxane (PDMS) membrane to suspend the magnet. The device had an overall volume of 2.25 cm^3^, and was able to produce 68.0 µW of power, leading to a power density of 30.22 µW/cm^3^. Although this result can be considered high compared to other harvesters of this scale, the excitation of 3 g acceleration and a resonant frequency of 108.4 Hz is impractical in real-world applications. Using a magnet as a proof mass in an MEMS harvester can drastically increase its size; this is due to the structure needed to housing the weight associated with magnets.

In [49], Sun et al. presented a unique electromagnetic energy harvester structure with an effective closed magnetic circuit. When the energy harvester was vibrating, a permanent magnet pair with an opposing polarity configuration caused the greatest change in the magnetic flux linkage in the solenoid.

**Figure 3:**
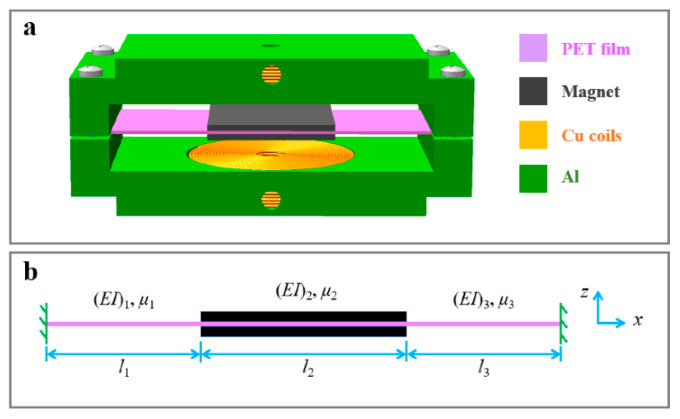
Lu et al. [50] proposed (**a**) a schematic diagram and (**b**) a vibration structure of a nonlinear electromagnetic vibration energy harvester (n-EVEH) based on a double-clamped beam.

A monostable double-clamped beam nonlinear electromagnetic vibration energy harvester was suggested in [50], as shown in Figure 3. A distributed parameter analytical model was created to assess output performance. It was discovered that while the frequency bandwidth widened, the nonlinearity of the double-clamped beam had no impact on the maximum output. In addition, as the excitation intensity was raised, the resonance frequency, frequency bandwidth, and maximum output all rose.

**Figure 4:**
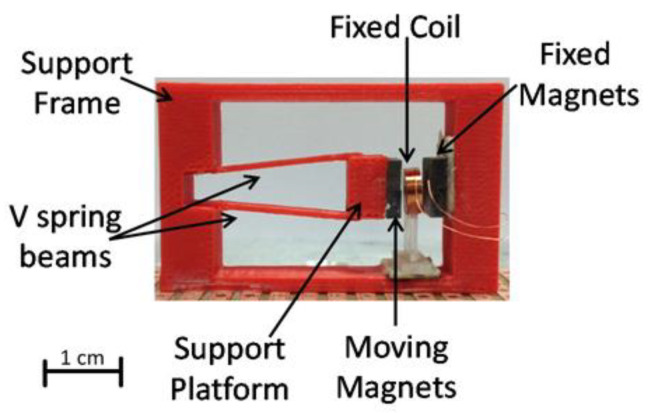
The 3D printed harvester created by [52]. A V-shaped spring is used to vibrate a magnet on one side, while the other uses a fixed magnet.

Palagummi and Yuan [51] developed an EVEH in the form of a moving magnet suspended by repulsive magnets. Their experiment led to a power output of 1.72 mW (rms) at a frequency of 2.1 Hz, with an excitation of 0.081 m/s^2^. Furthermore, they discussed the issues of eddy currents being produced in their device and possible ways in which they can be decreased, leading to a more efficient harvester. P. Constantinou and S. Roy [52] were able to create a non-linear harvester by implementing a 3D printed ‘V’ shaped spring structure, as illustrated in Figure 4. The harvester used a stationary coil and an immobile magnet on one side, while the ‘V’ supported a moving magnet on the other. The unique design allowed for a bandwidth of 3 Hz, ranging from 146–149 Hz. It was able to produce 2.5 mW of power with an acceleration of 1 g. The small size of the device (6 cm^3^) was achieved by only having one side of the magnetic structure move. Hence, a sufficient power density of 0.4 mW·cm^−3^ is achieved. This design demonstrates that a moving magnet is a viable option in the construction of an electromagnetic harvester.

### 4.2. Moving Coil Structure

**Figure 5:**
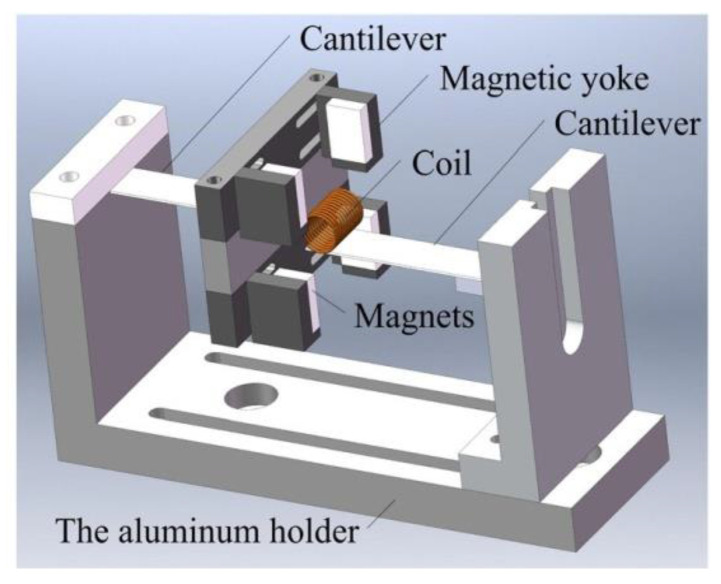
The design was proposed by [53], which uses a moving coil induced by 4 magnets. The design also has adjustable cantilevers for both the coil and magnets.

Moving coil systems often have the characteristic of high output voltage. This is due to the high number of turns needed for the proof mass. This was confirmed by Qiu et al. [53], who were able to achieve 9.04 V at 50.8 mW of power from an input of 14.9 Hz and 0.5 g acceleration with a coil of 1500 turns, as shown in Figure 5. It was observed that the peak power increased sharply as more turns were added to the coil. It was also proven that as each loop was added to the coil, the proof mass would gain a small amount of weight. This property allows the system to have a highly tunable resonance frequency.

Chen et al. [54] created an MEMS-style sandwich EVEH that utilized two cantilevers and a spring platform. This design is unique because both the two coils and the magnet are movable, which leads to several resonance frequencies being achieved. This larger bandwidth is ideal, as ambient vibrations commonly change due to environmental factors. The authors achieved resonances of 253 Hz, 330 Hz, and 430 Hz and produced peak-to-peak voltages of 172 mV, 104 mV, and 112 mV for the resonances, respectively.

**Figure 6:**
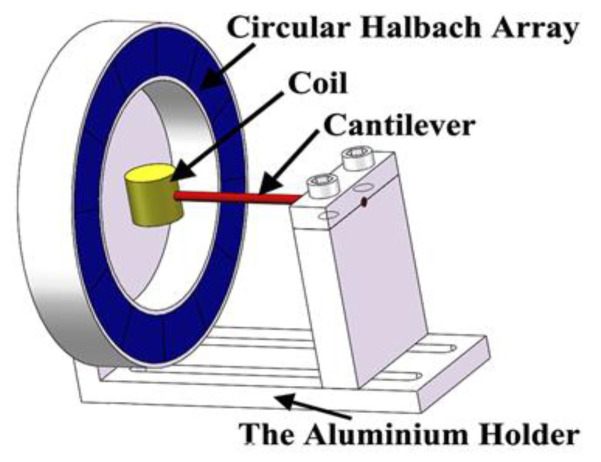
A circular Halbach array harvester that implements a moving coil via a cantilever [55].

Qiu et al. [55] experimented using circular Halbach arrays and a moving coil, as shown in Figure 6. They researched the increase in efficiency by using Halbach arrays, which could operate in multiple directions. The harvester was able to produce 7.29 mW of power when excited at a frequency of 15.4 Hz and an acceleration of 0.5 g. Qiu et al. varied the number of turns of the moving coil in an attempt to extract more power. They discovered that the power would decrease as the number of turns surpassed 700. They also concluded that the angle at which the harvester was vibrated showed an insignificant difference in the peak-to-peak output voltage. Thus, the design of a moving coil and circular Halbach arrays should be researched further.

## 5. Fabrication Technologies

Advances in fabrication technologies and materials have led to more efficient harvesters being built. Vibrational harvesters are generally produced in one of two ways, either by microelectromechanical systems (MEMS) processes or traditional mechanical fabrication. Mechanically fabricated harvesters are usually made with reasonably large magnets and a wound coil. Meanwhile, MEMS are manufactured using highly precise machines that use techniques such as lithography, micro electroplating, dry etching, deposition, etc. [56,57]. As most applications for EVEH require a compact design, MEMS technologies have been extensively researched and tested for fabricating energy harvesters.

### 5.1. Microelectromechanical Systems (MEMS) Technology

Peng et al. [3] constructed an MEMS harvester using lithography, KOH etching, silicon-silicon bonding, sputtering, PECVD, electroplating, ion beam, and DRIE etching techniques. These processes can be very tedious, but they offer great accuracy to the designs, leading to MEMS-based harvesters being able to have an accurate resonance close to their analytical modeling values. The authors’ final device, which consisted mainly of copper planar coils and a magnet, had a resonant frequency of 242 Hz and was able to generate 0.55 µW of power with a peak-to-peak voltage of 28 mV.

Seong et al. [58] developed an MEMS EVEH that investigated the properties associated with the choice of spring used. The proposed design was built using a two-legged spiral flat spring to mount the permanent magnet. This spring shape was chosen because it allowed for high flexibility, producing greater output. It also had the ability to carry a wider magnet, as well as having an easily tunable design. It was able to produce 270–437 µW of power over a large frequency range of 422–466 Hz.

Jo et al. [59] constructed an MEMS EVEH for the application of harnessing human vibrations. Human motion is typically associated with low-frequency and high-amplitude vibrations. To improve longevity, they utilized magnetic springs to help dampen the effects of large accelerations. In some mechanically fabricated harvesters, springs can wear and lead to fatigue cracks.

A hybrid technology of microelectromechanical systems (MEMS) and flexible circuits has been used to design and create an electromagnetic vibrational energy harvesting (EVEH) device [60]. The planned EVEH is made up of a stack of high-density flexible planar coils and a disc magnet sustained by four microfabricated silicon springs.

### 5.2. Traditional Technology

**Figure 7:**
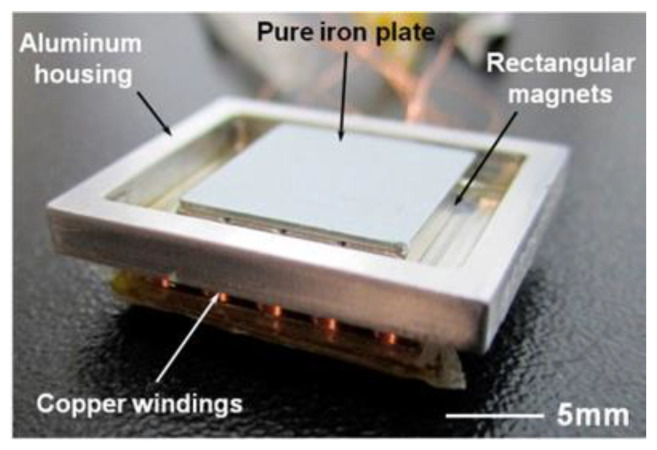
The device created by [62]. The aluminum housing allows the rectangular magnets to float on a thin layer of ferrofluid.

Traditionally, fabricated harvesters typically rely on mechanical fabrication techniques. Due to this, they are larger than MEMS-sized devices. Chae et al. [61,62] investigated the advantageous properties of ferrofluid as a lubricant in electromagnetic harvesters. The design of [62] used magnets floating on a thin layer of ferrofluid above coils, as shown in Figure 7. They ran a test of 93,600 cycles to view the effects of ferrofluid over a long harvesting period. It was observed that the structure without ferrofluid showed a decrease in power of 59.73%, whereas the ferrofluid design had a reduction of only 1.02%. It was also noted that the ferrofluid would cause less thinning of the magnet and casing, leading to a more controlled gap between the magnet and the coil.

The design of the harvester by Qiu et al. [63] implemented a coil attached to a cantilever beam, which undulated next to a Halbach array. Many traditionally manufactured devices place less emphasis on overall volume and more emphasis on output power. This leads to many of their designs having large numbers of turns. Qiu et al. demonstrated that very high voltages can be achieved. They were also able to demonstrate that an increase in cantilever length led to increased voltage. They proposed that this discovery was due to the increased amplitude of the coil. It was also shown that the diameter of the coil was important when designing a mechanically fabricated device. A steep drop-off in output voltage was observed, which was caused by a partial canceling of the magnetic flux through the coil.

Traditionally, fabricated harvesters can benefit from faster prototyping thanks to advancements in manufacturing techniques. This is demonstrated in [64], in which they developed a 3-D printed harvester. The design included a static coil and magnet, as well as a moving magnet on one side that was held in place by printed ABS plastic. Because of its shape and material properties, the plastic provided a spring mechanism. As a result, the prototype could produce 2.9 mW of power at 1 g acceleration. When excited at 1 g, the harvester had a bandwidth of 146–149 Hz and a power density of 0.48 mW/cm^3^. Mechanically fabricated harvesters are commonly researched since their construction machinery is more easily accessible than MEMS machines. MEMS holds the promise of real-world applications, but conventionally fabricated devices are commonly used as building blocks for new MEMS harvester designs.

## 6. Circuitry

The output from an electromagnetic harvester is generally an alternating current (AC), which is due to the nature of the poles from the magnet acting on the coil. This AC current must be converted into a direct current (DC) for it to be used by a suitable circuit. The simplest way to achieve this is by using a full-wave rectifier [65,66]. However, most of the voltages produced by electromagnetic harvesters are too low [67] to turn on the diode in the rectifier. Therefore, in most cases, the generated AC voltage must be boosted to a sufficiently high level for rectification. In general, there are two solutions for increasing the voltage of an electromagnetic harvester: multiple harvesters in series and the use of a Dickson charge pump. Using multiple harvesters in series allows the generated voltages to accumulate, which can be doubled, tripled, and more, depending on the number of cells used [68]. Thanks to their low power consumption, Dickson charge pumps are commonly used to multiply voltages. The final output can then be connected to the charging circuit of a battery or capacitor to be later used by the wireless device.

### 6.1. Full Wave Rectification

**Figure 8:**
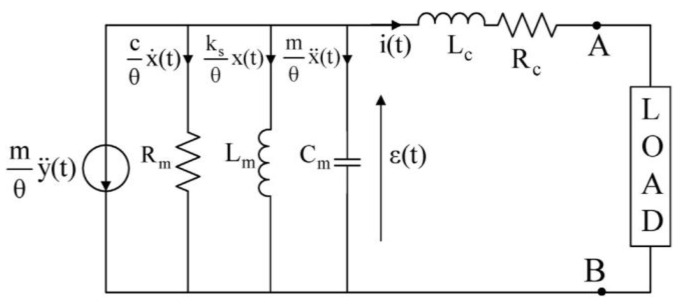
An analytical circuit model in [66], which analytically determined the effects of a bridge rectifier on an electromagnetic harvester system.

Full-wave rectification is an essential component in power conversion from AC form to DC form and is achieved by using diode bridges. Balato et al. [66] investigated a resonant electromagnetic vibration energy harvester (REVEH) to study the effects of a bridge rectifier on power output. Analytical models for a bridge rectifier were created and later confirmed by the harvester, as shown in Figure 8. The investigation into the maximization of extracted power led to the discovery that the power to the load is greatly dictated by the output voltage at the end of the bridge rectifier. The authors concluded that the insertion of a proper discrete reactive component increases the extraction of power from a REVEH loaded with a diode bridge rectifier.

**Figure 9:**
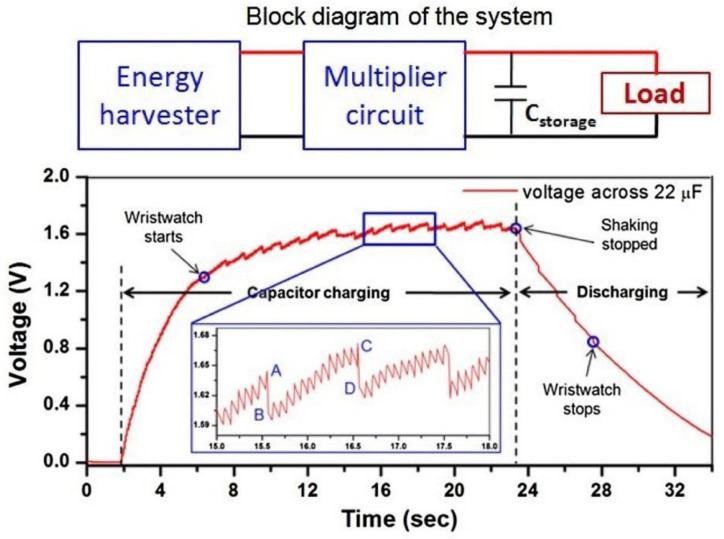
The basic circuit layout for the energy harvesting system described in [8], as well as the system’s capacitor characteristics during operation.

Halim et al. [8] constructed a harvester that was able to power a wristwatch. The system used human hand motion to generate power. It consisted of a cantilever beam with a magnet attached to one end. A circuit comprised of a full-wave rectifier and a voltage multiplier was implemented to charge a capacitor, as shown in Figure 9. This simple device and its experiments gave a strong foundation for the basic circuitry needed to power an ultra-low power (ULP) device. The experiment carried out by [1] used four Schottky diodes to rectify the current. A voltage drop of 0.15–0.45 volts was seen over the rectification circuit, which is significantly high in energy harvesting applications, as many harvesters have a voltage output lower than 500 millivolts. Hence, many harvesters are forced to use voltage multipliers [69].

### 6.2. Voltage Multiplication and Wireless Circuits

Voltage multiplication is used by a power management circuit to convert the low DC output to a higher voltage for its use. As some of these power management circuits require additional power to operate, a power supply is usually needed in addition to the harvester. This takes away from the idea behind the use of vibrational harvesters for energy harvesting purposes. Hence, Bryn Edwards et al. [70] developed an EVEH that also implemented a piezoelectric harvester to form a hybrid. The hybrid allowed for two resonant frequencies, but, more importantly, the piezoelectric harvester was able to produce a peak-to-peak voltage of 6 V and 10.5 µW of power. This additional source would allow for an electromagnetic output power of 34 µW and 18.5 mV (rms) to be converted to a high-voltage DC with the use of a low-power rectifier and voltage multiplier.

A harvester produced by Beeby et al. [71] achieved 51% efficiency when converting vibrations of 0.6 ms^−2^ acceleration at 52 Hz. The device was able to produce 58 µW (rms) with a volume of 0.8 cm^3^ and a weight of 1.6 g. This device has recorded the highest efficiency for any EVEH of this size. More astonishingly, Beeby et al. were able to power a wireless microsystem with the device. The wireless microsystem includes a power conversion circuit, energy storage, microprocessor, accelerometer, and an AM transmitter module. All these components were designed to be powered by 2.2 V or less. This allowed the charge pump to easily raise the initial voltage of 1.12 V from the harvester to the needs of the system. For the Dickson charge pump, through PSpice simulation, it was found that low-power Schottky diodes and 100 µF stage capacitors were optimal. The whole system was controlled by a MicroChip PIC16F676 microprocessor and was powered by a supercapacitor charged by the harvester. The PIC offered an analog-to-digital conversion for the accelerometer sensor and the ability to be used in low-power sleep mode. A major hurdle referenced in several reports is the trouble of operating an electrical circuit during a cold start. To combat this issue, a voltage-level detector was implemented.

The overall system of the device was built for power efficiency. The PIC periodically detects whether the capacitor has enough voltage to be able to power the circuit. Once a suitable level of power was reached, the accelerometer was powered, and 15 samples were taken. The microprocessor would then determine the peak value from the samples and send a signal to the receiver, which included a synchronization bit and identification byte, allowing for a more refined signal. This experiment is at the forefront of research and shows the highly applicable use of EVEH’s. It can be noted that applications will be exponential as the efficiency of EVEH’s and the circuits used by them are ever increasing.

## 7. Discussion

Many different designs in the literature in recent years have been implemented and trialed by researchers. Table 1 gives a comprehensive overview of various features and their outcomes on the harvested voltage and power.

As most applications require a small device as well as sufficient power output, the higher the power density (PD), the more applicable the device is. It was discussed in [72] that a power density of 2 mW·cm^−3^ is viable for a harvester to be used as a generator in the real world. The highest recorded power density was that of an MEMS device proposed by [58], which had a PD of 2.714 mW·cm^−3^. Although this device had a large bandwidth of 44 Hz over a range of 422–466 Hz, this resonance frequency is unfavorable in practice, as most ambient vibrations are well under 200 Hz. This means that the device would require some form of frequency-up conversion device, similar to that discussed in [35]. This addition would drastically increase the overall volume and cause a significant loss of power density. A more practical harvester design was created in [1], in which the PD was 1.552 mW·cm^−3^. It was also able to achieve the highest power in the literature. The design’s success was due to its large scale, which could implement magnetic stacks with air gaps between each magnet. However, this design shows a lack of scalability when compared to others.

Another parameter used to determine the merit of a design is the normalized power density (NPD), with a unit of mW/cm^3^/g. NPD includes the variable of acceleration in the overall power density. As many harvesters are evaluated at different excitation levels, NPD provides a better representation of a harvester’s efficiency, thereby being the deciding factor for all energy harvesters and their potential real-world applications.

It can be determined that a moving coil system results in a large output voltage. However, this type of design usually consists of a long cantilever, resulting in the design having scalability issues. It also leads to the design having a poor PD when compared to moving magnet devices. A trend for MEMS-fabricated harvesters can also be seen in the literature. The power densities for the MEMS devices are commonly in the tens of µW/cm^3^ range. This low value is due to problems with the scalability of the harvesters. Generally, the magnet and substructure are difficult to minimize and are typical of comprising most of the volume. More research is needed in the field of designing a more compact substructure and magnetic arrays. Once this is achieved, MEMS harvesters will be able to fill the gap in the current power harvesting.

## 8. Conclusions

From the first principles developed by Faraday, electromagnetic harvesters have undergone intense research into the advancement of self-sufficient wireless systems. Many designs have been investigated and trialed to build wider bandwidth devices to enhance efficiency, with some devices being able to generate power spikes over multiple frequencies. Meanwhile, other researchers have investigated the boost in magnetic field density by trialing multiple magnets and using various types and arrangements. The fundamentals of using a moving coil as opposed to a moving magnet have also been tested, leading to higher voltages being produced due to a larger number of turns. Advancements in other industries have also led to the evolution of MEMS-sized harvesters. Their small size offers promise for potential applications, whether they are wireless sensors or small electronic circuits.

## Figures and Tables

**Table 1:** Comparison of the characterizations in various designs of electromagnetic harvesters.

Reference	Moving Type	No. of Magnets	Fabrication	Frequency (Hz)	Acceleration (g)	Voltage (mV)	Volume (cm^3^)	Power (mW)	Power Density (mW/cm^3^)	Normalised Power Density (mW/cm^3^/g)
[34]	both	1	Traditional	19.4	0.019	910	125	2	0.016	0.842
[53]	both	4	Traditional	14.9	0.5	9004	-	50.8	-	-
[46]	coil	1	MEMS	391	0.122	-	0.29	9.6 × 10^−7^	3.3 × 10^−6^	2.7 × 10^−5^
[31]	coil	1	MEMS	1285, 1470, 1550	-	1.1–3.6	0.036 ^1^	4.5 × 10^−6^	1.25 × 10^−4^	-
[3]	coil	1	MEMS	242	0.5	28	-	0.55 × 10^−3^	-	-
[30]	coil	1	MEMS	100–800	-	0.01–0.13	0.32	16.01 × 10^−9^	0.5 × 10^−6^	-
[63]	coil	HA ^2^	Traditional	12.65	0.5	-	164	90.35	0.55	1.1
[55]	coil	HA	Traditional	15.4	0.5	2.08	-	9.32	-	-
[43]	coil	HA	Traditional	11.2	0.5	21,200	-	-	1.39	2.78
[42]	coil	comb	MEMS	400	-	-	0.28	3.12 × 10^−3^	0.011	-
[38]	magnet	1	Traditional	5.8	2	-	19.2	0.1036	0.0054	2.7 × 10^−3^
[73]	magnet	1	MEMS	3.33	1.26	-	0.763	0.1133	0.14849	0.118
[2]	magnet	1	MEMS	80	0.47	0.9	2.262	0.12	0.053	0.113
[51]	magnet	1	Traditional	2.1	0.008	-	-	0.00172	-	-
[58]	magnet	1	MEMS	422–466	-	-	0.161	0.437	2.714	-
[59]	magnet	1	MEMS	8	0.25	-	-	0.43	-	-
[39]	magnet	1	MEMS	55	14.9	18	0.13	6.1 × 10^−4^	4.69 × 10^−3^	3.15 × 10^−4^
[70]	magnet	1	Traditional	3 to 7	-	18.5	-	0.034 ^3^	-	-
[1]	magnet	2	Traditional	4	0.7	-	83.09	129	1.552	2.217
[20]	magnet	2	Traditional	30–80	-	-	-	0.4–3	-	-
[74]	magnet	2	Traditional	25.6	0.2	-	-	2.82	-	-
[8]	magnet	2	Traditional	-	Hand shaking	93.5	3.9	0.203	0.052	-
[28]	magnet	2	Traditional	5.17	2.06	-	6.47	11.89	0.33	0.16
[64]	magnet	2	Traditional	147–152	1	-	-	2.9	0.48	-
[45]	magnet	2	MEMS	371	13.5	46.3	1.008	-	0.02356	1.745 × 10^−3^
[48]	magnet	2	MEMS	108.4	3	88.8	2.25	0.068	0.03022	0.01
[52]	magnet	2	Traditional	146–149	1	-	6	2.5	0.4	0.4
[40]	magnet	2	Traditional	-	Walking	-	12.7	2.46	0.19	-
[16]	magnet	2	MEMS	78.43	-	1500	-	0.31537	-	-
[62]	magnet	4	Traditional	13	3	-	1.94	0.493	2.54 × 10^−4^	8.47 × 10^−5^
[75]	magnet	4	Traditional	36	0.5	-	-	0.109	-	-
[76]	magnet	6	MEMS	50	-	-	-	20.6	-	-
[41]	magnet	8	MEMS	-	Running	380	0.565	0.04316	0.076	-
[61]	magnet (ferrofluid)	4	Traditional	12	3	470	1.94	0.07126	0.037	0.0123

^1^ Doesn’t include magnet/support structure. ^2^ Halbach arrays. ^3^ Power output of 0.0445 mW when piezoelectric output is also considered.

## Data Availability

Not applicable.
